# A Systematic Review of Human Neuroimaging Evidence of Memory-Related Functional Alterations Associated with Cannabis Use Complemented with Preclinical and Human Evidence of Memory Performance Alterations

**DOI:** 10.3390/brainsci10020102

**Published:** 2020-02-13

**Authors:** Grace Blest-Hopley, Vincent Giampietro, Sagnik Bhattacharyya

**Affiliations:** 1 Department of Psychosis Studies, Institute of Psychiatry, Psychology & Neuroscience, King’s College London, London SE5 8AB, UK; k1334159@kcl.ac.uk; 2 Department of Neuroimaging, Centre for Neuroimaging Sciences, PO Box 089, Institute of Psychiatry, Psychology & Neuroscience, King’s College London, London SE5 8AB, UK; vincent.giampietro@kcl.ac.uk; 3 South London and Maudsley NHS Foundation Trust, Denmark Hill, Camberwell, London SE5 8AB, UK

**Keywords:** cannabis, memory, functional magnetic resonance imaging, THC, systematic review

## Abstract

Cannabis has been associated with deficits in memory performance. However, the neural correlates that may underpin impairments remain unclear. We carried out a systematic review of functional magnetic resonance imaging (fMRI) studies investigating brain functional alterations in cannabis users (CU) compared to nonusing controls while performing memory tasks, complemented with focused narrative reviews of relevant preclinical and human studies. Twelve studies employing fMRI were identified finding functional brain activation during memory tasks altered in CU. Memory performance studies showed CU performed worse particularly during verbal memory tasks. Longitudinal studies suggest that cannabis use may have a causal role in memory deficits. Preclinical studies have not provided conclusive evidence of memory deficits following cannabinoid exposure, although they have shown evidence of cannabinoid-induced structural and histological alteration. Memory performance deficits may be related to cannabis use, with lower performance possibly underpinned by altered functional activation. Memory impairments may be associated with the level of cannabis exposure and use of cannabis during developmentally sensitive periods, with possible improvement following cessation of cannabis use.

## 1. Introduction 

Cannabis is the most-used illicit drug worldwide [1], with many beginning to use it during their adolescent years [2,3]. Acute effects of the drug have been shown on cognitive performance, particularly in the domain of memory [4], with impairments being observed in all aspects of memory function, such as encoding, storage, and recall [5,6]. In addition to evidence about its acute effects, meta-analytic evidence has documented that long-term use of cannabis is associated with memory deficits [7].

Brain-structural alterations in cannabis users have been previously attributed to underlie deficits in memory performance. Reduced hippocampal volumes have been observed in cannabis users [8,9,10], with some studies showing evidence of a dose-dependant effect [11,12,13]. Along with this, cannabis users have shown volume reductions in the medial temporal cortex, particularly in the parahippocampal gyrus and temporal pole [14], as well as decreased cortical thickness in the orbital frontal cortex [14,15,16,17], frontal gyrus [17], and prefrontal cortex [18]. Other evidence suggests that structural alterations are not robust in the hippocampus [17,19,20,21], the orbitofrontal cortex [13,22,23], frontal gyrus [23], or prefrontal regions [17], or for overall grey matter volumes [19,24,25,26,27,28], even following meta-analysis [29]. Therefore, proposed cognitive deficits in cannabis users may be better explained by alterations in the functioning of relevant brain regions. 

The cannabinoid 1 (CB1) receptor is the main central cannabinoid receptor through which the leading psychoactive component of cannabis, delta-9-tertahydrocanabinol (THC), exerts its effect. The CB1 receptors are expressed ubiquitously throughout the brain [30], although higher densities are observed in regions key for memory functioning, such as the hippocampus and related medial temporal lobe structures and the frontal cortex [31]. Cannabis use may alter functioning of the key neural substrates involved in the processing of memory by affecting the homeostatic role of the endocannabinoid system, particularly when exposure occurs during developmentally sensitive periods [32].

Memory is a multidimensional construct and may be classified based on temporal characteristics into short-term (e.g., working) and long-term memory (e.g., declarative memory, i.e., the memory of facts and events; or procedural memory, i.e., the memory of skills or habits); its content (e.g., into verbal, visual, or spatial memory) or stage (e.g., encoding, consolidation, or retrieval) [33]. Declarative memory may be further classified into episodic or associative memory (i.e., memory for events and associations) and semantic memory (i.e., memory for meanings and facts) [34]. In the context of cannabis use, human neuroimaging studies have typically used cognitive paradigms involving working, associative, or spatial memory or encoding and recall stages [35]. Working memory requires the involvement of the prefrontal cortex, inferior and ventral temporal cortex, and the hippocampus [36,37,38], while spatial memory requires input from the hippocampus and prefrontal cortex [39], particularly for encoding [40]. Encoding into associative memory requires input from the hippocampus, medial temporal cortex, frontal cortex. and cingulate cortex [41,42,43,44,45,46], while recall of information relies on activation of the medial temporal cortex, including the hippocampus and parahippocampus, as well as the posterior parietal cortex and prefrontal cortex [47,48,49]. The hippocampus is therefore important in the context of multiple domains of memory processing and in both encoding and retrieval of information [50].

Previous work has reviewed both the cognitive [5,7,51,52,53,54] and neurofunctional [55,56,57,58] effects of cannabis, both acutely and chronically, in the context of memory processing. Although a number of systematic reviews have summarised brain-structural alterations [56,59,60] as well brain-functional alterations [56,59,60,61,62,63] more broadly over a wide range of cognitive domains associated with cannabis use, functional alterations in the context of memory processing in cannabis users have not been systematically and comprehensively summarized to include up-to-date literature [54]. Therefore, in order to summarise the current literature, we have conducted a systematic review of studies that have employed functional magnetic resonance (fMRI) techniques in conjunction with cognitive activation paradigms that involve memory processing, to investigate memory-related brain-functional alterations in long-term cannabis users (CU) compared to nonusers (NU). In addition, we review relevant preclinical and human studies investigating memory-related cognitive impairments (both cross-sectional and longitudinal) in association with nonacute cannabis or cannabinoid exposure, as well as human studies employing imaging techniques other than fMRI, to provide a comprehensive summary of current evidence linking the effects of persistent cannabis use on memory performance and brain functioning during memory processing. Furthermore, as the period of adolescence is thought to be a period of greater vulnerability to the effects of cannabis and cannabinoids [64,65,66,67], we also discuss the role of participant age (adolescent or adult) and age of onset of cannabis use as potential factors that may influence the extent of harm from cannabis use evident in current literature. We also link existing evidence to the effects of abstinence from cannabis exposure, as previous literature has documented the importance of this as a factor influencing the persistence of functional alterations associated with cannabis use [62,68]. Meta-analytic evidence focusing on memory performance in otherwise healthy recreational cannabis users suggests that cannabis use is associated with alterations in several memory domains, including prospective memory, working memory, verbal or visual memory/ learning/ recognition except for visual working memory, and visual immediate recall [7], suggesting that review of neuroimaging evidence should point toward altered activation in brain regions sub-serving these particular domains in cannabis users. 

## 2. Methods

### 2.1. Systematic Search of fMRI Studies

**Figure 1:**
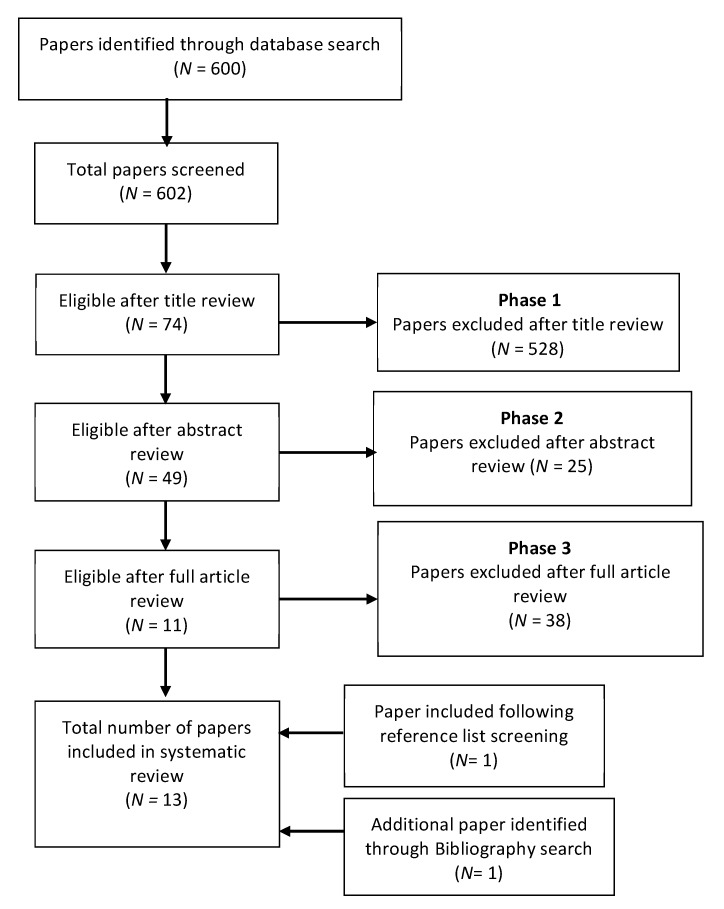
Identification of papers for systematic review.

A systematic search of previous studies comparing brain functional differences in CU and NU and employing fMRI in conjunction with memory processing tasks as activation paradigms was completed using the PUBMED database following the Cochrane Handbook [69] and the MOOSE guidelines [70]. We employed two categories of search terms: (1) those related to cannabis—cannabis, marijuana, marihuana, THC, and tetrahydrocannabinol—and (2) those related to neuroimaging technique: fMRI, imaging, functional activation, BOLD. The search was limited to human studies and was assessed for suitability through an initial screening of the titles, then abstracts, and a final full article review. An initial PUBMED search was completed on 21/10/2015 and was then repeated on 22/1/2020. Reference lists were also screened from included manuscripts and published reviews. Only manuscripts meeting the following criteria were included, as shown in Figure 1:

- Original peer-reviewed data-based publication, reported in the English language.
- Compared habitual, otherwise healthy cannabis users (>50 occasions of self-reported lifetime cannabis use) with healthy controls (<50 occasions of self-reported lifetime cannabis use).
- Used fMRI in conjunction with a memory-based cognitive activation task.

Studies were excluded if they did not use a cognitive activation paradigm or did not include a memory-based task; did not clearly indicate the extent of cannabis use in the cannabis user group; or involved use less than or equal to 50 times in their lifetime in the cannabis user group; were non-English-language studies.

### 2.2. Review of Other Evidence of Effects of Persistent Cannabis Use on Memory Performance (Preclinical and Clinical Evidence) and on Memory-Related Brain-Function Alterations Using Neuroimaging Modalities Other than fMRI 

Studies investigating memory performance in humans and animals and brain-function alterations related to memory processing using neuroimaging techniques other than fMRI in humans were identified through a bibliography search of previous systematic and narrative reviews [5,56,58,71,72]. To capture papers that have been published since the previous reviews, a search was carried out using the PUBMED database for relevant studies using the search terms “cannabis” or “marijuana” or “cannabinoid” and “memory”, which was completed on the 7/6/2018. These further papers were screened initially through a search of titles, then abstract, and finally a full article review. For the purposes of this review, we included only studies that used memory processing tasks with group comparison between cannabis or cannabinoid-exposed groups and a non-exposed or non-using control groups. Other studies that employed study designs different to this, but focused on the topics of interest in this review, have been discussed in the text, although they are not included in the tables. 

## 3. Results

### 3.1. Systematic Review of Human fMRI Studies Investigating the Association between Cannabis Use and Memory-Related Brain Function 

The initial search for fMRI studies comparing CU to NU while they performed memory-based cognitive activations tasks carried out in October 2015 identified 598 manuscripts. Of those, 10 met our inclusion criteria. Two further studies were identified by our final search on 22/1/2020 [73,74], and a further was identified from reference list screening [75]. Thirteen papers assessing memory-processing-related brain-activation differences between CU (*n* = 267) and NU (*n* = 261) using fMRI were identified in total. All included studies are reported in Table 1. Three of these papers involved only adolescents [76,77,78] CU (*n* = 72), NU (*n* = 79), while the remaining investigated adults CU (*n* = 195), NU (*n* = 182) with a group average age over 20 years. Four papers investigated spatial memory [73,78,79,80] and five associative memory [74,76,81,82,83], and four investigated working memory [76,84,85,86], while verbal learning [77] and false memory [75] were investigated by one paper each. Seven papers reported on group differences in whole-brain activation (WBA) [73,75,77,79,80,81,87] while eight investigated regions of interest (ROI) [76,77,79,81,82,83,84,85]. Four papers found CU to have performed worse than NU in the scanner-based memory task [75,79,81,83], nine found no significant performance difference [73,74,76,80,82,84,85,86,87]. 

#### 3.1.1. Summary of Results—Adult Studies 

Three studies investigated spatial memory in adults [73,79,80] using different types of tasks (water maze [79]; dot probe task [73,80]) and employed a whole-brain analysis approach. Opposite patterns of activation were identified in the superior and middle frontal gyri and putamen in two studies [79,80], while no difference was observed in a third study [73]. Tervo-Clemmens et al. included participants with low levels of cannabis use and long periods of abstinence, with only 15 of the 46 CU group having used in the previous year [73], which might explain the absence of difference between CU and NU in that study. Snieder et al. also employed an ROI analysis approach, finding only deceased activation in CU compared to NU in brain regions similar to their whole-brain analysis (WBA) approach, although they did not find any group difference in activation in the hippocampal ROI. 

Associative memory in adults was assessed by four studies [74,81,82,83]. Three studies investigated activation during learning, with two finding that activation decreased in CU in the frontal and temporal regions, with one using both WBA and ROI [81] and another only using the ROI [82] approach, although Nestor et al. found an opposite direction of activation in the parahippocampal gyrus. Blest-Hopley et al. found CU to have increased activation in the inferior, superior, and middle frontal gyrus bilaterally and in the right medial frontal gyrus in a WBA. During recall of information, a decrease in activation was seen in two studies in the anterior cingulate cortex [82,83], but no group difference was found by another [74]. Carey et al. found activation decreased in other regions, including the hippocampus, using ROI analysis during a paired location number task, where CU had more repeated errors [83]. 

Two studies investigated working memory [84,85], where both studies employing ROI analysis found no difference in activation between CU and NU. 

Using a task used to investigate brain activation associated with false memory, Riba et al. [75] found CU not only had more false memories but also decreased activation compared to NU in temporal, parietal, and frontal cortex, as well as thalamus, caudate, and precuneus, employing a whole-brain analysis approach. 

Only one study found activation differences in the hippocampus [83] during the recall condition of an associative memory task, where CU had decreased activation compared to NU, whereas another found no significant differences using an ROI analysis approach [79] during a spatial memory task. Parahippocampal activation was, however, seen to be decreased in CU compared to NU during spatial and associative memory tasks [79,82], although another study found parahippocampal activation increased in CU compared to NU while performing an associative memory task [81]. The majority of studies reporting activation differences between groups found activation to be decreased in CU compared to NU in a variety of memory tasks [75,79,81,82,83]; however, some found regions of increased activation [74,80,81], with many regions overlapping with areas previously found as having decreased activation. Three studies, however, found no differences between CU and NU, using both whole-brain (WBA) and ROI analysis approaches [73,84,85].

Finally, a study not meeting our entry requirement for cannabis use levels compared 18- to 22-year-old cannabis users, based on their use over the previous 3 months, with those who had not used over that period. Using a visual memory task, no difference in activation was seen in the ROI of the IFG and hippocampus during the encoding condition; however, WBA found CU had decreased activation in the cerebellum (left), insula, basal ganglia, superior frontal gyrus, right precentral gyrus, and bilateral parahippocampal gyri. During the recognition condition of the task, ROI analysis showed CU had significant decreased activation in the hippocampus bilaterally and left IFG, while WBA revealed that CU had decreased activation in the cerebellum (bilateral), insula, basal ganglia and cingulate, and left posterior parietal cortices [88]. A longitudinal fMRI study of working memory from a baseline to 3 years in heavy cannabis users found that activation of the working memory network remained stable [89] over time despite continued moderate to heavy use of cannabis as well as nicotine, alcohol, and illegal substances.

#### 3.1.2. Summary of Results—Adolescent Studies

Of the studies in adolescent cannabis users, one used a spatial working memory test and reported decreased activation in frontal and parietal regions in adolescent CU compared to NU [87]. Another study used an associative picture task, finding no significant difference in activation between adolescent CU and NU in ROI analysis [76]. Jager et al. also investigated working memory in adolescents using a letter recognition task and, using ROI analysis, found increased activation in CU compared to NU in frontal and parietal regions [76]. A third study of adolescent CU found no differences in brain activation during verbal encoding following both WBA and ROI analysis [77]. Of the two studies reporting activation differences between groups, both found activation in the superior parietal lobe to be increased in CU using different forms of working memory tasks, though opposite patterns of activation were seen in the dorsolateral prefrontal cortex by these studies [76,87]. 

### 3.2. Human Studies Investigating Memory-Related Brain Function Alterations Using Neuroimaging Modalities other than fMRI 

Only two studies have employed neuroimaging techniques other than fMRI to investigate neurofunctional differences between CU and NU in the context of memory processing. Battisti et al. [90] investigated event-related potentials (ERP) during a verbal memory task wherein participants’ responses were characterised based on whether they correctly recalled (CR) or did not recall (NR). In this study with 24 participants (CU = 24; NU = 24; average age of CU 36.4 (11.2) and NU 35.5 (11.5)), CU had an average of 17 years of near-daily use and had all used in the week prior to testing, with a minimum of 13 h between last use and testing. They identified attenuated latency in the frontal region of CU compared to NU in N4, a window around 350 ms, thought to originate in the hippocampus during encoding [91]. The amplitude of frontal and parietal zones was decreased in CU. The NR latency was attenuated in line with longer periods of cannabis use. Block et al. [90] investigated cerebral blood flow using positron emission technology (PET) during delayed and novel recall tasks in 18 CU who reported daily use of cannabis for over 2 years prior to recruitment and underwent 26 h of monitored abstinence and compared them with 13 NU. They found a decrease in frontal blood flow in CU compared to NU, which was most prominent whilst recalling newly presented words. Differences between CU and NU included the fact that language-based memory-related activity in the left hippocampus was observed to be higher in NU, with CU lacking this lateralization of hippocampal activation. 

### 3.3. Human Studies Investigating Association between Cannabis Use and Memory Performance Alterations—Cross-Sectional Studies 

Seventeen cross-sectional studies were identified that investigated the effects of cannabis use on memory performance by comparing CU and NU using various cognitive tasks engaging different domains of memory (Table 2). Twelve studies investigated adult cannabis users and five investigated adolescent participants. 

#### 3.3.1. Summary of Results—Adult Studies

Four studies employed a verbal learning task where stimuli were visually presented, finding that CU performed significantly worse at recall of words [93,96,97,98]. However, in the study by Wadsworth and colleagues, this was only observed in CU that had used in the 24 h prior to testing [96]. Pope et al. found in both studies that CU performed worse compared to NU at verbal memory test over the first week of examination following an abstinence of 0, 1, and 7 days, but by day 25, CU only performed worse on long-delay recall [97,98]. In contrast, no difference in performance was seen in a smaller former CU group compared to NU on the verbal memory test [98].

Auditory verbal learning tasks were used by six studies, where word lists were read out to the participants. CU had worse recall performance than NU [94], with higher performance deficits seen in those who had used for longer periods [99,107] or at a higher dose [95]. While Rodgers et al. tested participants after a month of self-reported abstinence from cannabis use, they did not carry out testing of urine or blood to confirm abstinence [94]. McKetin et al. did not report time since last use but interestingly found that abstinence did not improve performance at two waves of four-year retesting [95]. No difference between CU and NU was seen in recall performance in two studies [92,100]. However, Cengel et al. found that CU performed worse compared to NU on five of the eight conditions tested, including false recall and maximum and total learning [92] after three days’ abstinence. 

Three studies used the California verbal learning test [101,102,103], with two finding that CU performed significantly worse than NU [102,103], and the third study no significant difference [101]. Levar et al. only found a significant difference in the long-delay cued recall condition out of four tests of short and long delay free and cued recall with earlier-onset users performing worse than late-onset users [102], and Schuster et al. found that CU performed significantly worse at encoding and recall only in early-onset users, i.e., before the age of 16 [103]. It is unclear whether duration of abstinence or extent of cannabis use may have accounted for the difference in results in these three studies. While Levar et al. studied participants with an average abstinence of a few days, participants in the study by Schuster et al. were only required to be abstinent on the day of testing. In contrast, the study by Gruber et al. [92] required only a 12-hour abstinence period of their participants but failed to detect significant performance difference between users and nonusers, although their participants reported the highest mean years of cannabis use of these three studies. 

Using the Wechsler memory scale, Pope et al. compared a set of heavy-using CU to NU at four time-points of abstinence, finding no significant difference between CU and NU after 25 days of abstinence [97], replicating findings of their previous smaller study [98]. Rodgers et al. also used a test for general memory, finding significant impairment in their CU group after abstinence for 1 month compared to NU [94]. 

None of the four studies investigating visual memory found a significant difference between CU and NU [94,97,98,101]. 

#### 3.3.2. Summary of Results—Adolescent Studies

Two studies investigated auditory verbal learning in adolescent CU compared to NU [104,105]. Solowij et al. observed these deficits were in line with the quantity and frequency of cannabis used, as well as the age of onset of use, which remained even after controlling for premorbid intellectual ability [104]. In contrast, Hanson et al. found that performance in CU returned to a level comparable to NU after a 3-week period of abstinence, though users in this study had comparatively low levels of cannabis use [105]. 

The California verbal learning test was used by two studies with one finding some deficits in adolescent CU compared to NU following a 1 month abstinence [106], while the other found no significant difference following a 3- to 11-month period of abstinence [12]. Medina and colleagues found deficits were trend level in CU (with cannabis use ranging between 60 and 1800 times per lifetime) compared to NU in the California verbal learning test and Wechsler Memory scale Logical memory test of first recall, immediate, and delayed recall and recognition scores, while there were no impairments in verbal list learning and visuospatial memory [106]. 

Both immediate and general memory performance was tested by Fried et al. in current heavy (average 12.4 (9.8) joints per week), light (<5 joints per week), and former cannabis users (over 3 months abstinence), with all three groups compared to NU separately. Heavy CU performed worse in both immediate and general memory performance, whereas light CU and former CU had no significant difference in performance compared to NU [108]. 

Two studies investigated working memory in adolescent CU compared to NU [105,108], with one finding CU performed significantly worse initially, which was no longer evident after 3 weeks of abstinence [105]. These results were consistent with evidence from another study reporting significantly impaired immediate and delayed memory in current heavy cannabis users but not in light users or in former users [108]. 

One study investigated spatial learning performance following a 1 month abstinence in a group with cannabis use ranging between 60 and 1800 times per lifetime, with no significant difference in performance between CU and NU [106]. 

### 3.4. Human Studies Investigating Association between Cannabis Use and Memory—Longitudinal Studies 

We identified six studies that used some form of longitudinal study design to investigate whether memory deficits seen in CU predated the use of the drug or developed following cannabis use. In one of the earliest reports, Fried et al. controlled for differences in cognitive performance prior to initiation of drug use and compared immediate, general, and working memory performance between heavy CU and NU. Heavy CU performed significantly worse in all memory domains compared to NU. Immediate and general memory impairments persisted after controlling for pre-drug-use performance, though working memory performance was no longer significantly impaired after controlling for pre-drug-use performance [108]. In an 8-year follow-up study, Tait et al. found that cessation of use in heavy cannabis users was associated with significant longitudinal improvement in immediate recall performance compared to continued heavy cannabis users [109]. In another cohort study, Meier et al. measured IQ at the age of 13 years old and used it to control for memory performance at a follow-up age of 38 years. After also controlling for years of education, cannabis use was found to be significantly associated with decline in memory performance [110]. A 25-year follow-up study by Auer et al. found that after excluding current CU and adjusting for potential confounders such as baseline memory performance cumulative lifetime exposure to cannabis was strongly associated with poorer performance subsequently in a verbal memory task in a dose-dependant manner [111]. However, another study employing a longitudinal design did not find any significant adverse effect of cannabis use on longitudinal change in performance in memory tasks at 4 and 8 years follow-up in an older (40–46 years) cohort of participants [95]. In contrast, in another study, Castellanos-Ryan et al. found a bidirectional relationship between cannabis use and cognitive performance such that poorer short-term memory and working memory performance at age 13 (prior to initiation of cannabis use) was associated with earlier age of onset of cannabis use, and earlier onset, and more frequent cannabis during adolescence, in turn, was associated with neurocognitive decline by age 20 [112]. However, a specific effect of cannabis use on subsequent memory performance was not reported in this study.

### 3.5. Preclinical Studies Investigating the Effect of Cannabis Use on Memory

A total of 18 animal studies were identified in our search (listed in Table 3). Exposure times to cannabinoids ranged from 14–180 days, while washout periods ranged from 0–116 days. All studies presented used rats. Thirteen studies investigated spatial memory, eleven investigated short-term memory, and four examined working memory. Six studies treated two separate groups of animals with cannabinoids during either adolescence and adulthood. 

Spatial memory, investigated using learning maze-based tasks, was been found to be impaired in rats following chronic exposure to THC or CB1 receptor agonist by five studies [120,121,123,124,126]. Five other studies using maze tasks found that previous exposure to cannabinoids did not have a significant effect on performance [113,118,125,127,128]. Object location tasks have also been used to assess spatial memory in rats, with impairments found in two studies following a prolonged cannabinoid exposure [122,123], but not in two others [114,115]. 

Impairments have also been observed in short-term memory in eight studies [113,114,115,116,117,119,122,123], although these findings have not been replicated in three other studies [121,128,129]. Two studies reported deficits in working memory performance following cannabinoid exposure [123,126], although two other studies did not find cannabinoid exposure having a significant negative effect in working memory performance [115,119]. No effect was seen on recognition or operant learning in rats following adolescent exposure [129].

Of the studies that exposed the animals during adolescence to adulthood, four found that cannabinoids had a negative effect on memory performance in the adolescent group, but not in the adult group in the same study [116,117,119,122]. Two studies, however, found no difference between the two age groups and did not find any deficit following chronic cannabinoid exposure at all [127,128]. 

Investigating memory performance after a period of washout is useful to disentangle the residual effects of cannabinoids from any acute effects. Of the four studies that included a memory test done within 24 h of the last drug administration, all reported deficits in the cannabinoid-treated animals [114,115,123,124]. Impairments in spatial memory appeared to reverse after an abstinence of a few days in two studies [123,126]; however, they were still present at 75 days abstinence in short-term memory tasks in one [123]. Rats who showed deficit following WIN 55,212-2 exposure in short-term memory after a 24 hr abstinence had no significant differences to controls after a 51 day washout period [115]. Similarly, those that had shown poorer performance after THC exposure in spatial memory at 24 h had no significant differences after 25 days [124]. Memory deficits remained following washout periods of 30 [120] and 116 [113] days.

## 4. Discussion

Our objective was to carry out a comprehensive review of alterations in brain functioning and performance in the domain of memory associated with persistent cannabis use, by drawing upon evidence from human and relevant preclinical studies. To this end, first, we carried out a systematic review of studies investigating brain-functional alterations in CU compared to NU using fMRI. We also reviewed published studies that have employed neuroimaging techniques other than fMRI to investigate memory-related functional alterations associated with cannabis use. Finally, to situate this understanding within the context of specific subdomains of memory affected, we performed two focused narrative reviews of studies investigating alterations in memory performance associated with persistent cannabis use employing a range of designs: studies in humans using cross-sectional and longitudinal designs to compare CU and NU and preclinical studies comparing cannabinoid exposed animals with nonexposed animals. These results are discussed under separate subsections below.

### 4.1. Systematic Review of fMRI Studies Using Memory Tasks

Our systematic review of functional brain activation during memory performance found CU to have altered brain activation, although no consistent pattern emerged either in terms of the direction of alteration in activation or the brain regions affected, although changes appeared to be mostly focused in the frontal and temporal regions. Altered activation in the hippocampus was found in some studies, particularly those employing ROI analysis approaches focusing on the hippocampus, but without a conclusive direction of change. Alteration of hippocampal activation was perhaps less frequently observed than one would have expected considering the central role of the hippocampal region in memory processing. Activation was altered more often during the encoding/learning stage than while recalling information, similar to previous evidence investigating memory performance [130].

Of the three studies reporting hippocampal activation differences between CU and NU during learning, there was no consistent direction of change [81,82,83], despite having similar ages of participants as well as ages of onset and levels of cannabis use in their participant groups. Inconsistencies in abstinence periods could have played a role in these differences, as cannabis users in the study by Nestor et al. had a self-reported abstinent range of 2–45 h, while those in the study by Carey et al. had an average of 101.67 h of self-reported abstinence, and Jager et al. required subjects to have tested negative for THC on urine screening indicating that the psychoactive substance was no longer present in their system. A further study investigating hippocampal activation with ROI analysis approach found no difference in brain activation between CU and NU during encoding but decreased activation in CU compared to NU during recognition. WBA found the parahippocampal gyrus to have significantly less activation during encoding in CU compared to NU [88]. 

The level of previous cannabis use might have been a potential confounder in studies that have reported no significant differences between CU and NU. Significant differences in activation between CU and NU were observed in studies using similar tasks investigating CU with high levels and prolonged periods of cannabis use [74,76,80,87] but not in those with lower levels and/or less extensive cannabis use [73,84]. Counter to this, however, was that continued cannabis use was not associated with altered activation patterns during working memory at 3-year follow up in another study [89]. Although a study with a wide range of cannabis use levels in the previous three months found higher levels of cannabis use was correlated with decreased hippocampal activation during recognition [88], it should be noted that no abstinence period prior to scanning was reported for cannabis use. Differences in task performance may also have contributed to the differences seen in activation; however, task difference during fMRI was only found in four of the studies [75,79,81,83].

The literature on brain activation differences measured by fMRI between CU and NU during memory tasks still lacks clarity, possibly due to heterogeneity of both the extent of cannabis use as well as the quantity, frequency, and age of onset of cannabis use, which have been found to correlate with alterations seen in cannabis users [88,104,131]. In addition, the wide range of tasks employed measuring different domains of memory function (e.g., spatial or working memory) mean it is difficult to perform a robust meta-analysis of their results or to draw consistent conclusions between studies. Subgroup analysis in a previous meta-analytic study focusing only on memory tasks in adult cannabis users found only decreased activation in the inferior frontal gyrus, pre- and post-central gyrus and precuneus, although the previously mentioned inconsistencies and the modest number of studies available for this analysis were major limitations [61]. In the fullness of time, meta-analysis of well-matched studies focusing on a particular domain of memory, such as verbal memory, spatial memory, etc., may better serve to reveal a consistent pattern of functional alterations in the context of memory processing associated with cannabis use. 

Functional activation differences between CU and NU were more consistently seen in adult populations of cannabis users than in adolescents, although a previous meta-analysis of all cognitive domains has identified functional difference in CU compared to NU in both adults and adolescents [61,63]. Lack of differences in brain activation between adolescent CU and NU may be attributable to the design of some adolescent studies, with participants having used cannabis for a shorter period of time and therefore not having been exposed to the threshold at which functional differences become detectable using fMRI, and also simply the smaller subset of studies available. For example, one study in adolescent users that found no difference between CU and NU in whole-brain or ROI analysis during verbal learning had four groups of participants with and without alcohol abuse as well as with and without CU, meaning that comparisons of CU to NU groups without alcohol abuse involved a relatively small number of participants. CU participants also had a relatively long period of abstinence, and their cannabis use levels were relatively modest [77], indicating that certain functional differences may become less evident with longer periods of abstinence. This was further supported from a comparison of adolescent CU with recent cannabis use (2–7 days of abstinence) and a group of CU following a longer period of abstinence (27–60 days of abstinence) performing a spatial working memory task, suggesting that duration of abstinence may have an impact on alterations in functional activation associated with cannabis use [86]. More recent CU had greater activation compared to abstinent users in the bilateral insula and superior frontal gyrus; right—inferior gyrus; left—precentral gyrus, medial and middle frontal, and gyrus. In contrast, abstinent users only had greater activation compared to recent users in the right precentral gyrus, which may reflect a compensatory response in recent CU requiring recruitment of additional brain regions compared to abstinent CU in order to perform the memory task, as has been suggested previously [80]. 

Acute effects of THC confounding previous literature cannot be ruled out in some studies that have investigated participants who were not confirmed to have a negative result for THC on urine drug screening and/or studied participants after only short periods of abstinence from cannabis. Although a sustained period of abstinence may have alleviated some of the group differences in functional activation, abstinence periods did not consistently predict the detection of group differences in functional activation. While this may suggest that certain functional alterations are more robust than others and therefore detectable in CU even following a washout period of the drug, it is very likely that this also reflects the possibility that functional alterations observed cross-sectionally are not just attributable to the effects of drug exposure but an interplay with baseline differences between CU and NU that predate initiation of cannabis use. 

### 4.2. Review of Cross-Sectional Human Studies Investigating Memory Task Performance 

We reviewed studies of both adults and adolescents, comparing the performance of memory tasks between CU to NU. Verbal memory performance was negatively affected by cannabis use [93,94,96,97,98,102,103,104,105,106], with increased deficits associated with cannabis use levels [95,99,107]. This was also shown in a study of cannabis users ranging from light to heavy use after a month abstinence [132], in both adolescent and adult users, though deficits in performance were not reported in all studies [12,97,100,101]. Visual memory did not appear to have been affected in CU [94,97,98,101], although there were only a limited number of studies that investigated this paradigm, which were all conducted only in adults. General memory performance, as tested in adults and adolescents, was also negatively affected by cannabis use [94,108]. Working memory, in contrast, was shown to be affected in some studies, but these effects were not sustained following abstinence from the drug [96,108]. Spatial memory was only tested in one study in adolescents and was found to be unaffected by cannabis use [106].

Evidence from some studies suggests that longer duration of cannabis use may have an adverse impact on memory performance [93,107] with the amount of cannabis used correlating with performance in some studies [95,106], implying that high levels and longer use of cannabis are related to reduced memory performance. An increased likelihood of memory impairments was found to be associated with an earlier age of onset of cannabis use [103,133], although this association may also have been a result of the longer duration of exposure in those that started earlier and therefore the greater amount of cannabis that they had been exposed to and not necessarily an effect of earlier age of onset of use per se. This issue was investigated in one study that found significant deficits in early-onset users compared to late-onset users, despite late-onset users having used for longer periods [103], suggesting that there is very likely a developmentally sensitive period when the effects of cannabis exposure on memory performance are more prominent in humans, consistent with preclinical evidence [116,117,119,122]. However, another study did not identify differences between early- and late-onset CU, although they also failed to detect any significant difference between all CU and NU [101], perhaps due to a large range in years of cannabis use.

Abstinence from cannabis use reversed some memory deficits observed, with an earlier meta-analysis showing no significant effect of the drug after a 4-week abstinence on performance [68]. We also observed in our review, which included a number of studies published subsequent to that meta-analysis, that the interval period from last cannabis use to the assessment of task performance may have a bearing on the likelihood of studies reporting poorer memory performance in CU compared to NU. This may reflect two different factors influencing task performance in cannabis users in these studies: the residual acute effects of cannabis influencing memory task performance in studies involving shorter periods of abstinence, as well as the recovery of CB1 receptor density following an initial downregulation after prolonged exposure to cannabis, resulting in absence of detectable differences in memory performance in studies involving longer periods of abstinence [134]. Lack of significant difference in memory performance between CU and NU, as found in several studies with abstinence periods from as little as 3 weeks, may mean there is a reversal of the negative effects of cannabis use on memory performance. 

It is of interest to note that the effort made during memory tasks has also been found to be negatively associated with frequency of cannabis use [135,136]. Therefore, it is possible that the effort made in completing the tasks may have also influenced the relationship between cannabis use frequency and performance deficits observed in learning and memory tasks. There is some evidence that the extent of impairments in task performance is not always perceived fully by cannabis-using participants [137], which may also adversely affect their effort during the task, thereby influencing their performance.

### 4.3. Review of Longitudinal Human Studies Investigating Memory Task Performance

While some longitudinal studies of memory performance suggest that CU may be associated with a deficit in memory performance following prolonged use, the evidence is not unequivocal, especially when baseline cognitive ability predating initiation of cannabis use is taken into consideration. McKetin conducted a follow-up study finding that CU performed the same irrespective of whether they had continued to use or had ceased use [95]. This may indicate that cannabis use does not contribute to memory decline in a linear fashion and continued use past a critical sensitive neurodevelopmental period may no longer be associated with continuing decline in memory performance, especially as participants in McKetin’s study were middle-aged individuals. Of course, one cannot completely rule out the possibility that cannabis use does not have a direct causal effect on poor memory performance or indeed of a bidirectional effect. 

Although poorer premorbid memory performance may be partly attributed in some users to their lower cognitive attainment in the domain of memory performance, improvement in performance observed following the cessation of cannabis use suggests that cannabis use may in fact have a direct deleterious effect. Further longitudinal studies are needed to tease apart the effects of cannabis use from other genetic and environmental effects. Other studies have aimed to address the question of the causal nature of the relationship between cannabis use and memory performance and have employed study designs that allowed them to account for genetic and other environmental confounders. A monozygotic twin study by Lyons et al. [138] investigated the effects of cannabis use on memory in 54 pairs of twins. They reported only a trend level decrement in performance in the CU group compared to nonusers during recall of a verbal learning task. During other memory tasks, no group differences were detected. However, participants in this study had a wide range of exposure to cannabis, with 37% of the CU using less than 52–300 times in total lifetime. Furthermore, CU participants included in the study were only required to have used cannabis regularly for one year in total with no restriction as to how long ago the period of use was. In many subjects, they had ceased to use regularly for an average of 27 years, with all subjects having at least a 1-month period of abstinence from cannabis when they were tested [138]. Another twin study by Meier et al. found that greater cannabis use by one twin was associated with poorer working memory performance compared to their nonusing twin, in the absence of any difference in their IQ at baseline. However, such a difference was not observed for the other memory (spatial memory) and executive (visual processing) tasks [139]. While cannabis use has been found to be associated with lower intelligence scores, another study employing a discordant twin design reported that family traits were more associated with intelligence performance [140]. Collectively, these studies provide some further evidence suggesting that cannabis use may have a causal role in memory deficits observed in cannabis users, although whether the deficits persist following abstinence from use remains debatable, and genetics may play a larger role in determining memory performance. 

### 4.4. Review of Preclinical Studies Investigating Memory Task Performance

We found that animal studies have investigated the effect of not only extracts directly obtained from dried cannabis plants with varying potency in earlier experiments [113,120], but also analytical grade plant-derived cannabinoids such THC, or synthetic cannabinoids such as the CB1 receptor agonists WIN and CP55940 [141]. The discrepancy in results between studies may reflect difference in dose, duration of exposure, experimental conditions, animal species used, and the precise periods of exposure during adolescence or adulthood. Both age of onset as well as the washout period following exposure to cannabinoids appeared to influence whether deficits in memory performance were detected following chronic exposure to cannabinoids. However, in two studies using cannabis extracts in adult rats for a prolonged period of 90 and 180 days, deficits were still detectable even after a washout period of 116 and 30 days respectively [113,120]. This may suggest that, although adolescence acts as a critical period for exposure and abstinence periods may be associated with decrease in observed deficits and a possible reversal of residual effect, duration of exposure is also a critical factor that may influence the extent and persistence of deficits. Age of onset also appeared to be a critical factor as exposure to cannabinoids during the early adolescent phase produced a significant impairment in learning, compared to the late adolescent period, when no significant effect was seen [116,117,119,122]. 

The period of adolescence in rat models is short and so makes it difficult to administer drugs for long enough periods of time when targeting them in the developmental period. However, many studies did attempt to administer drugs for the majority of the adolescent period and saw significant results. The lengthier period of adolescence in humans allows for much longer exposure during neuronal maturation and possibly presents an extended period during which cannabis may have a greater detrimental effect on cognition. Preclinical studies with shorter periods of administration of drugs found fewer deficits in memory, but the period of washout from drug administration seemed to have a major influence on the outcome of studies. 

The hippocampus is a key region for memory performance [142], and animal studies have been useful to investigate structural, functional, and histological effects of chronic cannabinoid exposure here. In rats, following exposures to THC and WIN, even after a washout period, alteration in dendrites of hippocampal pyramidal neurons has been found [143,144]. Interestingly after a washout period, synaptic density was not different in drug-treated animals, even though hippocampal volume and structure were found to be decreased in rats [143,145]. In monkeys administered cannabis and THC chronically, both structure and function in the hippocampus were found to have changed, and there were also synaptic changes [146], suggesting that alterations in hippocampal structure and function may underlie functional and performance differences. 

Behavioral tests in animals have not provided conclusive evidence of memory deficits following cannabis use, although structural and histological investigations have shown robust evidence of cannabinoid-induced changes. Collectively, the body of research completed in animals investigating the effects of chronic cannabis use and its possible effects on memory function appears limited in comparison to that investigating the acute effects of cannabinoids. 

## 5. Conclusions 

To summarize, cannabis use has been shown in some studies to negatively affect memory-related brain functioning and task performance, particularly verbal memory and encoding in human studies, with preclinical evidence generally consistent with human evidence. Effects have also been observed in recall and working memory tasks, though these findings have been less robust. However, existing evidence regarding the effect of cannabis use on memory function is far from unequivocal, as evident from the heterogeneity in conclusions from the different studies. 

From the evidence reviewed above, three clear factors emerge that may underlie differences in results seen in studies of memory function following cannabis use. Firstly is abstinence/washout period from last use, which seems to vary a lot in all the animal and human studies reviewed. Longer abstinence periods do appear to be associated with a less pronounced difference between CU and NU participants and cannabis-exposed and -unexposed animals, although not in all cases, particularly following a long exposure. Collectively, results from human studies reviewed here showing recent cannabis use being associated with alteration in memory-related functional activation, which becomes less prominent following periods of abstinence and longitudinal data [89], suggest that cannabis users may compensate for neurophysiological deficits associated with drug use by recruiting a network of additional brain regions.

Secondly, cannabis use parameters, such as use of higher quantities, longer periods of use, and more regular use, appear to increase the chances of detecting differences in memory-related outcomes. Some, but not all, studies do report linear relationships with time and quantity of cannabis used. Future studies should therefore aim to systematically investigate associations with these parameters of cannabis use and also incorporate other parameters, such as type of cannabis used, that have been shown to be associated with other health outcomes [147]. Interestingly, some studies over extended periods of cannabis use suggest a plateau in observed changes from cannabis use, where alteration may only be linear in the initial period of use or only during developmental periods. 

Finally, the age at which cannabis use starts seems to be another important determinant, with animal literature in particular providing robust evidence for adolescence being a period of higher risk of brain alterations from cannabinoid exposure. From a biological perspective, this is a period of neuronal developmental processes, including brain development and altering binding affinity of CB1 receptors [64,65,66,67]. From a more social perspective, cannabis use during this period may result in poorer educational outcomes that may then in turn exacerbate memory performance impairments [148]. Cannabis use acutely impairs memory performance [149] and alters memory-related brain function [150] and may therefore adversely affect educational attainment. This is consistent with evidence showing that CU have an increased chance of leaving school earlier [151] and have poorer educational achievements [152] compared to NU. Pope et al. [133] also showed in their study comparing early- and late-onset users that there was a significant difference in the completion of a 4-year college course rates, with only 32% of early-onset CU compared to 60% of late-onset CU and 82% NU completing.

The detrimental effects of cannabis may be due to CB1-receptor-mediated disruption of hippocampal plasticity, a finding supported by animal histological investigations. Although there is a lack of evidence of significant effects on hippocampal activation from human studies, changes in hippocampal cerebral blood flow and ERPs [90,93] as well as structural differences [29] have been observed in CU compared to NU. Functional activation in other brain regions that express a high density of CB1 receptors were also found to be altered during memory processing tasks in cannabis users, possibly due to disruption of the normal functioning of the endocannabinoid system as suggested by overlap between brain regions with high CB1 receptor distribution [31] regions showing altered functioning in cannabis users (regions highlighted in [35]). 

One limitation inherent to studies investigating chronic cannabis users, as in any studies investigating recreational drug users and that affects this systematic review as well, is the bias associated with retrospective recall of usage pattern and type of cannabis consumed over time. This is particularly important as the longer-term effects of cannabis use may depend on the specific usage pattern as well as type of cannabis, particularly the ratio of different cannabinoids, which may have often opposing effects on brain function and connectivity [153,154,155]. Future investigations should therefore focus on employing prospective designs in conjunction with more accurate ways of quantifying cannabis use, perhaps using a similar model to alcohol units, in order to improve the quality of future studies [156].

To conclude, evidence summarized here suggests that memory performance deficits may be related to cannabis use, with lower performance in memory tasks possibly underpinned by altered functioning of a wide network of brain substrates that may result from changes at the synaptic level. This review did not summarise how functional connectivity may be altered in cannabis users, as was discussed in a previous review [35], which reported altered connectivity in cannabis users during cognitive task performance. However, further studies of functional connectivity, particularly during memory processing tasks, are necessary in order to understand how the coordinated activity of brain networks may be affected rather than just brain regions. Further research is also necessary, taking into account baseline cognitive performance or ability prior to initiation of cannabis use in order to conclusively establish whether these changes persist or are indeed reversible following cessation of cannabis use and to fully understand the potential determinants of reversibility such as period of use and the quantity or length of time of exposure to cannabinoids. Such granular understanding is necessary to inform public health policy to help mitigate harm from cannabis use in those that are most vulnerable.

## Figures and Tables

**Table 1:** Systematic review of cannabis users and nonusing control comparisons, using functional magnetic resonance imaging.

Study	Task	No. NU and Age	No. CU and Age	Age of Cannabis Use Onset (Years)	Abstinence Before Scan	Cannabis Use Levels	Results		
							Whole Brain Analysis	Region of Interest	Task Performance
Adult studies									
Carey et al., 2015 [82]	Associative Memory	15 22.8 (2.9) (SD)	15 22.7 (4.2)	15.97 (0.42)	101.67 (37.45) h	6.43 (1.07) years 7341.40 (2340.80) lifetime uses	N/A	Decreased in the dACC and left hippocampus during processing of error-related and re-encoding or correct response in CU.	Decrease in error recall and correction rate in CU and poorer learning from errors.
Cousijn et al., 2014 [83]	Working Memory	41 22.0 (2.3) (SD)	32 21.4 (2.4)	18.9 (2.3) Onset of Heavy Use	24 h	2.5 (1.9) Years 1619.5 (1428.9) Lifetime uses	N/A	No significant difference found in the task-defined working-memory network.	No significant difference
Jager et al., 2006 [84]	Working Memory	10 23.3 (0.95) (SD)	10 22.4 (1.11)		7 days	7.1 (3.9) years 1300 [675–5400] lifetime uses	N/A	No significant difference found in learning or recall for both tasks.	No difference in task performance for both tasks.
Jager et al., 2007 [81]	Associative Memory	20 23.6 (3.9) (SD)	20 24.5 (5.2)		7 days	1900 [675–10150] lifetime uses	N/A	Decrease activation in bilateral parahippocampal regions and R DLPFC during learning for CU. Decreased activation in the right ACC in CU during recall.	No difference in task performance.
Kanayama et al., 2004 [79]	Spatial Working Memory Task	12 27.8 (7.9)	10 37.9 (7.4)		6–36 h	[5100–54000] lifetime use	Increased activation in; R SFG, IFG, STG, PCG, putamen; bilaterally, ACG, MFG, caudate. Decreased activation in bilateral MFC in CU.	N/A	No significant difference
Nestor et al., 2008 [80]	Associative Memory	14 24.1 ± 1.3 (SEM)	14 24.4 ± 1.4	16.5 ± 0.4	2–45 h	5.7 ± 0.6 years	Decreased activation in R STG, SFG, MFG, and L SFG during learning for CU. No difference in recall.	Increased in activation in R parahippocampal gyrus during learning for CU. No difference in recall.	Lower level of recall performance in CU.
Riba et al., 2015 [74]	False Memory Task	16 37.6 (11.8)	16 37.6 (10.8)	17 (12–20)	28 days	21 years [3–39] 42000 lifetime uses average	Decreased activation in CU in the R temporal cortex and precuneus; left, DLPFC, thalamus, caudate and medial temporal lobe, bilateral parietal cortex when recognising false memories over correct.	N/A	CU showed significantly more false memories.
Sneider et al., 2013 [78]	Spatial Memory	18 22.8 (5.0) (SD)	10 20.3 (3.6)	15.6 (1.2)	12 hs	4 (2.4) years	Decreased activation in CU frontal pars triangularis, bilateral inferior frontal pars orbitalis, bilateral MFG, right pallidum and R putamen.	Decrease activation of R parahippocampal gyrus and cingulate gyrus in CU for recall motor control. No difference found in the hippocampus.	Similar performance, although CU showed more deficit in memory retrieval
Tervo-Clemmens et al., 2018 [72]	Spatial working memory	15 28.16 (0.71)	46 28.22 (0.72)	15.14 (2.27)	All THC negative	0.367 (0.683) mean joints per day—Only 15 used in the last year	No Significant Difference	N/A	No significant difference
Blest-Hopley et al., 2020 [73]	Associative Memory	21 24.24 (4.11)	22 24.95 (3.56)	14.67 (1.98)	12 h	6.19 (1.20) days per week 10.29 (3.10) years	Increased activation of bilateral; SFG, IFG, MFG, right medial FG in CU during encoding. No significant difference in Recall	N/A	No significant difference
Adolescent studies									
Jager et al., 2010 [75]	Working Memory and Associative Memory	24 16.8 (1.3) (SD) [13–19]	21 17.2 (1.0) [15–19]	13.2 (2.3)	5.1 (4.2) weeks	4006 (7555) lifetime uses	N/A	Increased activation in the IFG, SPC and PCC/DLPFC of users during novel working-memory task. No group difference in associative memory task.	No difference in task performance.
Schweinsburg et al., 2008 [77]	Spatial working memory Task	17 17.9 (1.0) (SD)	15 18.1 (0.7) (SD)		28 days	480.7 (277.2) lifetime uses 4 (1.6) years	Increased activation R superior parietal lobe, decreased activation R DLPFC in CU.	N/A	No significant difference
Schweinsburg et al., 2011 [76]	Verbal Encoding Task	22/16 17.6 (0.8) 18.1 (0.7) (SD)	8/28 18.1 (0.9) 18.0 (1.0)	14.5 (2.5) 14.9 (3.4)	117.6 (153.9) 43.4 (37.1)	426.5 (280.1) 517.6 (451.3) lifetime uses	No significant difference	No significant activation in hippocampus.	No significant difference

NU = non-using cannabis group, CU = cannabis-using group, parentheses () used for SD, square brackets [] used for range.

**Table 2:** Memory performance studies comparing cannabis users to non-using controls.

Study	Task	No. NU and Age	No. CU and Age	Age of Cannabis Use Onset (years)	Abstinence Period	CU Levels	Results	Notes
Adult studies								
Gruber, Sagar, Dahlgren, Racine, and Lukas, 2012 [92]	Rey-Osterrieth Complex Gigure (visual memory)	28 24.32 (6.65)	34 22.76 (6.57)	15.53 (2.16)	12 h	7.24 (7.30) years 19.24 (19.58) smokes per week	No significant difference seen between CU and NU	Had an early- (<16 years ) and late- (> 16 years) onset group. No significant difference was seen between early- and late-onset in either task
	California Verbal Learning Test						No significant difference seen between CU and NU	
Battisti et al., 2010 [90]	Verbal Memory Task	24 35.5 (11.5)	24 36.4 (11.2)	15 [12,13,14,15,16,17,18,19,20,21,22,23,24,25]	20 h (mean)	20.2 (9.7) years 30 [4–30] days per month	CU recalled significantly fewer words, which had a marginal correlation with the duration of use.	
Wadsworth, Moss, Simpson, and Smith, 2006 [93]	Immediate free recall task (episodic memory)	85 26.79 (4.64)	34 24.03 (5.28)			7.63 years [1–20]	No significant difference seen between CU and NU	This study was carried out in a workforce population
	Delayed free recall task (episodic memory)						No significant difference seen between CU and NU	
	Delayed recognition memory task (episodic memory)						No significant difference seen between CU and NU	
	Verbal reasoning task (working memory)						CU performed significantly worse; however, when cannabis use was considered for the last 24 h, this deficit was in line with the last use of cannabis	
	Semantic processing task						No significant difference seen between CU and NU	
Solowij et al., 2002 [94]	Rey Auditory Verbal Learning Test	33 34.8 (11.1)	ST—51 28.7 (5.5) LT—51 42.1 (5.2)	15.3 (2.6)	12 h	ST—10.2 [2.7–17] years 28.3 (5.2–30) days per month LT—23.9 [17.3–31.7] years (27.4 (3.5–30) days per month	LT—recalled fewer words and learned slower than both controls and ST—correlating with duration of use. ST- did not differ from controls	ST = Short term user LT = Long term user
	Rey Auditory Verbal Learning Test—long recall						All CU performed worse than controls overall; however, LT—recalled significantly less than before the delay time than ST—and NU	
Quednow et al., 2006 [95]	Rey Auditory Verbal Learning Test	19 23.42 (4.30)	19 21.42 (5.77)		3 days	6.55 (3.67) years 3.89 (4.72) times per week	No significant difference seen between CU and NU	This study mostly looking at an MDMA group. Did not have a high-using CU group
Pope, Gruber, Hudson, Huestis, and Yurgelun-Todd, 2002 [96]	Benton Visula Retention Test (visual memory)	87 40 [34–45]	77 36 [32–43]		0–28 days (month-long trial)		No significant difference seen between CU and NU on the test carried out on day 0, 7, and 28	
	Buschke’s Selective Reminding Test (verbal memory)						CU had significantly poorer performance at day 0, 1, and 7. At day 28, these differences no longer met significance, except in the long-delay condition	
	Wechseler Memory Test				28 days		No significant difference seen between CU and NU	
Pope, Gruber, Hudson, Huestis, and Yurgelun-Todd, 2001 [97]	Benton Visula Retention Test (visual memory)	72 39.5 [34–44]	63 36 [32–41]		0–28 days (month long trial)	19 [15–24] years smoking >6 joints per week	No significant difference seen between CU and NU	A second former heavy CU group (*n* = 45) was recruited with < 12 times use in the last month
	Buschke’s Selective Reminding Test (verbal memory)						CU had significantly poorer performance at day 0, 1, and 7. At day 28, these differences no longer met significance, except in the long-delay condition.	The former users showed no difference from controls on any task.
	Wechseler Memory Test						No significant difference seen between CU and NU	
Rodgers, 2000 [98]	Verbal Memory	15 32 [26–39]	15 30 [27–43]		1 month	4 days per week	CU performed significantly worse than NU	They did not test CU for abstinence
	Visual Memory						No Significant difference seen between CU and NU	
	General memory						CU performed significantly worse than NU	
	Delayed Recall						CU performed significantly worse than NU	
McKetin, Parasu, Cherbuin, Eramudugolla, and Anstey, 2016 [99]	Immediate Recall	4986 42.6 (1.5)	106 42.7 (1.4)			At least weekly	CU was related in a dose-related fashion to performance	
	Delayed Recall						CU was related in a dose-related fashion to performance	
Cengel et al., 2018 [100]	Immediate Memory	48 27.00 (6.19)	45 28.84 (6.37)	18.06 (3.95)	3 days	10.32 (6.12) years	No significant difference seen between CU and NU	
	Maximum Learning						CU scored significantly lower than NU	
	Number of repetitions						CU had significantly more/poorer performance than NU	
	Total Learning						CU performed significantly worse than NU	
	Recall Score						No significant difference seen between CU and NU	
	Recognition Scores						No significant difference seen between CU and NU	
	False Learning						CU had significantly more/poorer performance than NU	
	False Recall						CU had significantly more/poorer performance than NU	
Levar, Francis, Smith, Ho, and Gilman, 2018 [101]	California Verbal Learning Test	22 21.59 (1.94)	19 20.58 (2.52)	16.21 (1.69)	2.79 (3.10) days	4.37 (1.67) years	CU had worse performance, but only significant in the long-delay cued recall.	
Schuster et al. 2016 [102]	California Verbal Learning Test	48 21.5 (2)	27 19.6 (2.1)	15.1 (0.96)		2.9 (1.7) days per week 3.8 (2.1) years	CU performed significantly worse at encoding and recall than NU	Early-onset cannabis-using group (<16 years of age)
			21 21.2 (1.8)	17.8 (0.83)		2.9 (1.6) days per week 5.5 (1.7) years	No significant differences	Late-onset cannabis-using group (>16 years of age)
Adolescent studies								
Ashtari et al., 2011 [12]	California Verbal Learning Test	14 18.5 (1.4)	14 19.3 (0.8)	13.1 [9–15]	6.7 months [3–11]	5.3 (2.1) years	No significant differences	
Solowij et al., 2011 [103]	Rey Auditory Verbal Learning Test	62 18.07 (0.48)	52 18.67 (0.82)	15 [10–17]	12 h	2.36 (1.17) years 13.87 [0.5–30] days per month	CU recalled significantly fewer words than NU	
	Rey Auditory Verbal Learning Test—long recall						CU recalled significantly fewer words than NU	
	Word Recognition Test						CU recognised significantly fewer words that NU	
Hanson et al., 2010 [104]	Hopkins Verbal Learning Test	21 17.4 (1.0)	19 18.1 (0.8)	15.6 (1.6) regular weekly use	3.3 (3.2)	16 (9.2) days past month 465 (294.5) life-time use episodes	CU performed significantly worse than NU	
	Verbal Working Memory						CU performed significantly worse than NU	
Medina et al., 2007 [105]	California Verbal Learning Test	34 17.86 (0.99)	31 18.07 (0.87)		30 days	2.91 (2.08) years of weekly cannabis use	CU performed at trend level (*p* < 0.10) worse than NU	
	Verbal Story Memory						CU performed at a trend level (*p* < 0.10) than NU. Performance correlated with cannabis use	
	Verbal List Learning						No significant difference seen between CU and NU	
	Visuo-spatial Memory						No significant difference seen between CU and NU	
Fried, Watkinson, and Gray, 2005 [106]	Immediate memory	59 17.7 (0.7)	19 current light CU < 5 joints a week 18.0 (1.2)	15.7 (1.7)		1.8 (2.0) years	Current light CU did not differ significantly from NU on all three memory tasks	Three groups of CU in the study, completed all three of the tasks.
	General Memory		19 current heavy CU > 5 joints a week 17.8 (0.8)	15.0 (1.5)		2.6 (1.3) years	Current heavy CU performed significantly worse to NU at general and immediate memory, but not working memory	
	Working Memory		16 former CU 17.9 (1.1)	14.3 (1.3)		2.2 (1.4) years	Former CU did not differ significantly from NU on all three memory tasks	Former users had no regular use for 3 months

NU = Nonusing cannabis group, CU = Cannabis using group, parentheses () used for SD, Square Brackets [] used for range.

**Table 3:** Animal studies using memory tasks to investigate exposure to exogenous cannabinoids.

Study	Task Used	Age of Exposure	Washout Period	Animal Type	Drug Used	Results	Other Notes
Renard, Krebs, Jay, and Le Pen, 2013 [113]	Object Recognition	29–50 PND	28 days	Wister Rat	CP55,940	Drug-treated animals spent less time exploring novel objects and had significantly different times exploring familiar objects to control	Wister Rats had a larger effect of memory performance following drug exposure than Listerhooded Rats
	Object Recognition	29–50 PND	28 days	Listerhooded Rat	CP55,940	Drug-treated animals spent less time exploring novel objects and had significantly different times exploring familiar objects to control	
	Object Recognition	70–91 PND	28 days	Wister Rat	CP55,940	No difference in time exploring novel objects and familiar objects to control	
	Object Recognition	70–91 PND	28 days	Listerhooded Rat	CP55,940	No difference in time exploring novel objects and familiar objects to control	
	Object location	29–50 PND	28 days	Wister Rat	CP55,940	Drug-treated animals did not show a significant change to novel exploration time, where control did	
	Object location	29–50 PND	28 days	Listerhooded Rat	CP55,940	Drug-treated animals did not show a significant change to novel exploration time, where control did	
	Object location	70–91 PND	28 days	Wister Rat	CP55,940	Drug-treated animals showed no difference in behaviour to control	
	Object location	70–91 PND	28 days	Listerhooded Rat	CP55,940	Drug-treated animals showed no difference in behaviour to control	
Kirschmann, Pollock, Nagarajan, and Torregrossa, 2017 [114]	Object Recognition	34–54 PND	0 days	Sprague Dawlet Rats	WIN55,212-2	Drug-treated animals showed significant effect of drug on object recognition	
	Working Memory test	34–54 PND	17 days	Sprague Dawlet Rats	WIN55,212-2	No significant effect of drug on working memory performance	
	Object Location	34–54 PND	17 days	Sprague Dawlet Rats	WIN55,212-2	No significant effect of drug on object location test	
Harte and Dow-Edwards, 2010 [115]	Active Place Avoidance Testing	22–40 PND	33 days	Sprague Dawlet Rats	THC	Drug-treated animals performed worse than control	
	Active Place Avoidance Testing	41–60 PND	16 days	Sprague Dawlet Rats	THC	No significant effect of drug seen in performance	
Schneider and Koch, 2003 [116]	Object Recognition	40–65 PND	20 days	Wister Rat	WIN55,212-2	Drug-treated animals showed significantly impairment of recognition memory	
	Object Recognition	>70 PND	20–25 days	Wister Rat	WIN55,212-2	No significant effect of drug seen in performance	
O’Shea, Singh, McGregor, and Mallet, 2004 [117]	Object Recognition	30–51 PND		Wister Rat	CP55,940	Novel object recognition was significantly lower in drug-treated animals to controls; however, delay time had no significant effect between groups.	
	Object Recognition	56–77 PND		Wister Rat	CP55,940	No effect of treatment was seen between groups.	Only nine animals in the adult 56-77 PND-treated group
Rubino et al., 2008 [118]	Elevated Plus-Maze	35–45 PND	30 days	Sprague Dawlet Rats	THC	No significant effect of drug seen in performance	
Schneider, Drews, and Koch, 2005 [119]	Object Recognition	15–40 PND	45 days	Wister Rat	WIN55,212-2	No significant effect of drug seen in performance	
	Progressive Ration/Operant learning	15–40 PND	35 days	Wister Rat	WIN55,212-2	No significant effect of drug seen in performance	
Abush and Akirav, 2012 [120]	Water Maze	45–60 PND	24 h	Sprague Dawlet Rats	WIN55,212-2	Drug-treated animals took longer to find the platform	
	Water Maze	45–60 PND	10 days	Sprague Dawlet Rats	WIN55,212-2	A significant difference was found compared to rats tested at 24 h abstinence.	
	Object Location	45–60 PND	24 h	Sprague Dawlet Rats	WIN55,212-2	Drug-treated animals showed impaired long-term memory to control animals	
	Object Location	45–60 PND	10 days	Sprague Dawlet Rats	WIN55,212-2	Drug-treated animals showed impaired long-term memory to control animals	
	Object Location	45–60 PND	30 days	Sprague Dawlet Rats	WIN55,212-2	Drug-treated animals showed impaired long-term memory to control animals	
	Object Recognition	45–60 PND	24 h	Sprague Dawlet Rats	WIN55,212-2	Drug-treated animals spent significantly less time exploring novel objects	
	Object Recognition	45–60 PND	10 days	Sprague Dawlet Rats	WIN55,212-2	Drug-treated animals spent significantly less time exploring novel objects	
	Object Recognition	45–60 PND	30 days	Sprague Dawlet Rats	WIN55,212-2	Drug-treated animals spent significantly less time exploring novel objects	
Fehr, Kalant, and LeBlanc, 1976 [112]	Closed Field Maze	14 day treatment	24 h	Rats	THC	Drug-treated animals performed worse than control animals	
	Closed Field Maze		25 days	Rats	THC	No significant difference was seen between the groups	
Hill, Froc, Fox, Gorzalka, and Christie, 2004 [121]	Water Maze	15 day treatment	During daily treatment	Long-Evans Rats	3-11-Δ8-THC	Drug-treated group took much longer to learn the task, showed similar performance	
	Water Maze, plus time delay				3-11-Δ8-THC	Drug-treated animals had significantly worse performance	
Mateos et al., 2011 [122]	Spontaneous Alternation (Short-term memory) Task	28–43 PND	24 h	Wister Rat	CP55,940	Drug-treated animals performed significantly worse than control	
	Object Location	28–43 PND	37 days	Wister Rat	CP55,940	No significant effect of drug seen in performance	
	Object Recognition	28–43 PND	43 days	Wister Rat	CP55,940	Drug-treated animals performed significantly worse than control	
Rubino et al., 2009 [123]	Passive Avoidance	35–45 PND	30 days	Sprague Dawlet Rats	THC	No significant effect of drug seen in performance	
	Radial Maze	35–45 PND	30 days	Sprague Dawlet Rats	THC	Drug-treated animals had significantly more errors and took significantly more time to learn the maze layout	
Stiglick and Kalant, 1982 [124]	Radial Maze	180 days	30 days	Wister Rat	THC and CBN	Drug-treated animals made significantly more errors and less correct responses and took longer to learning the overall task	
Stiglick and Kalant, 1985 [125]	Radial Maze	90 days	31 days	Wister Rat	THC, CBN and CBD	No effect of drug was seen between groups	
	Avoidance test	90 days	116 days	Wister Rat	THC, CBN and CBD	Drug-treated animals performed worse than controls	
Cha, Jones, Kuhn, Wilson, and Swartzwelder, 2007 [126]	Water Maze	30–51 PND	28 days	Sprague Dawlet Rats	THC	No effect of drug was seen between groups	
	Water Maze	70–91 PND	28 days	Sprague Dawlet Rats	THC	No effect of drug was seen between groups	
Cha, White, Kuhn, Wilson, and Swartzwelder, 2006 [127]	Water Maze—Spatial task	34/36 PND + 21 day	28 days	Sprague Dawlet Rats	THC	No effect of drug was seen between groups	
	Water Maze—Non-Spatial Task	34/36 PND + 21 day	28 days	Sprague Dawlet Rats	THC	No effect of drug was seen between groups	
	Water Maze—Spatial task	69/74 PND + 21 days		Sprague Dawlet Rats	THC	No effect of drug was seen between groups	
	Water Maze—Non-Spatial Task	69/74 PND + 21 days		Sprague Dawlet Rats	THC	No effect of drug was seen between groups	
Higuera-Matas et al., 2009 [128]	Object Recognition	28–38 PND	63 days	Wister Rat	CP55,940	No effect of drug was seen between groups	
	Water Maze—Reference memory	28–38 PND	67 days	Wister Rat	CP55,940	No effect of drug was seen between groups	
	Water Maze—Spatial task	28–38 PND	67 days	Wister Rat	CP55,940	No effect of drug was seen between groups	

PND = Postnatal day, 3-11-Δ8-THC = 3-(1,1-dimethylheptyl)-(–)-11-hydroxy-Δ8-tetrahydrocannabinol, THC = Δ-9-tetrahydrocannabinol, CBN = cannabinol, CBD = cannabidiol.
